# Perioperative Venous Excess Ultrasound Score (VExUS) to Guide Decongestion in a Dilated Cardiomyopathy Patient Presenting for Urgent Surgery

**DOI:** 10.7759/cureus.20545

**Published:** 2021-12-20

**Authors:** Keevan Singh, Randall Carvalho

**Affiliations:** 1 Anaesthesia and Intensive Care Unit, Department of Clinical Surgical Sciences, University of the West Indies, San Fernando, TTO; 2 Anaesthesia and Intensive Care Unit, San Fernando General Hospital, San Fernando, TTO

**Keywords:** perioperative medicine, hepatic vein doppler, pocus, portal vein doppler, vexus

## Abstract

Venous excess ultrasound score (VExUS) is a recently described ultrasound-based scoring system that quantifies systemic congestion using Doppler flow indices of the hepatic and portal vein in addition to inferior vena cava assessment. There are many potential and emerging applications of this modality. We discuss the case of a severely congested heart failure patient presenting for urgent non-cardiac surgery where VExUS parameters were used to monitor and guide his decongestive therapy postoperatively.

## Introduction

Managing fluid balance in heart failure patients presenting for surgery is an ongoing challenge to the anesthesiologist. Often, these patients may require adjustment of their diuretic dose due to varying fluid requirements during the surgical period. Factors to consider include the clinical state of the patient, ongoing surgical losses and the stress response of surgery that may lead to volume retention. Fluid overload may lead to pulmonary edema and acute kidney injury (AKI) during the perioperative course in these patients. Similarly, excessive diuresis can result in hypoperfusion and also contribute to AKI [1].

Venous excess ultrasound score (VExUS) is a recently described approach that can assess patients with clinically significant organ congestion [2]. VExUS focuses on Doppler analysis of hepatic and portal veins in addition to standard inferior vena cava (IVC) assessment. IVC assessment has many limitations, which, of relevance in our patient, includes right ventricular dysfunction and varying breathing patterns [3]. VExUS parameters are easily obtained and provide an objective marker from which the clinician can adjust intravenous fluids or decongestive therapy [4]. The case discussed here highlights a novel application of VExUS in the intraoperative and postoperative period to individually tailor therapy in an advanced heart failure patient.

## Case presentation

A 49-year-old male presented with a large incarcerated inguinoscrotal hernia. He had recently been diagnosed as having dilated cardiomyopathy with an ejection fraction of 20%. Although he had poor exercise tolerance (<4 metabolic equivalents), his condition was clinically stable and managed on oral furosemide, carvedilol and enalapril. On admission to the hospital, he was clinically assessed as dehydrated and his diuretics were held. On preoperative assessment by the anesthesiologist, a point-of-care ultrasound (POCUS) was performed and he was noted to have global right and left ventricular dilatation with poor ventricular contraction. His IVC diameter was >2.1 cm with <50% collapsibility. The patient also complained about significant dyspnea on lying flat in bed. He was noted to have a blood pressure of 140/105, a heart rate of 95 bpm, and a respiratory rate of 20 breaths per minute with an oxygen saturation of 97% on room air. While conservative treatment of his hernia was attempted, the patient received diuresis with intravenous furosemide for the next four days. Furosemide dose ranged from 80-120 mg/day.

Once a decision for surgery was made, after failing conservative treatment, the patient was brought to the operating room where another POCUS was performed by the anesthesiologist using a handheld device, GE Vscan^TM^ (GE Healthcare, Waukesha, WI, USA) (Video 1).

**Video 1:** Preoperative point-of-care ultrasound

Despite receiving approximately four days of diuresis, the patient’s IVC size was still >2.1 cm with <50% collapsibility. On further assessment, a visibly pulsatile portal vein was also seen on color Doppler (Video 2).

**Video 2:** Preoperative VExUS Transthoracic echo views were performed using standard windows. The portal vein was visualized using the phased array probe laterally in the right upper quadrant. The hepatic vein was visualized in the standard subcostal window with the phased array probe. The approach to VExUS has been previously described [2]. VExUS, venous excess ultrasound score

Epidural anesthesia was planned and due to the likelihood of hemodynamic instability, no further diuretics were given despite the patient still complaining of significant orthostatic dyspnea.

Anesthesia proceeded via a slowly titrated lumbar epidural inserted at the L4/L5 level. An arterial line was inserted in addition to standard intraoperative monitors, and a low-dose noradrenaline infusion was preemptively started to maintain stable hemodynamics (<.1 ug/kg/min). The patient remained hemodynamically stable for the course of the surgery and was transferred to the intensive care Unit (ICU) for further care. An epidural infusion of 0.125% bupivacaine and fentanyl was started and continued for postoperative pain for 24 hr. Due to the preoperative VExUS assessment of portal vein pulsatility, a furosemide infusion of 20 mg/hr was given for a total of 4 hr. This yielded a large diuresis of approximately 4500 mL over 12 hr and significant relief of his dyspnea. VExUS was repeated after 12 hr and showed a non-pulsatile/phasic portal vein, a hepatic vein with reduced regurgitant flow, and a now collapsible though still enlarged IVC (Video 3).

**Video 3:** Postoperative VExUS VExUS, venous excess ultrasound score

The patient spent approximately two days in the ICU and was restarted on his maintenance diuretics after the completion of the initial furosemide infusion. He was successfully discharged home approximately seven days after surgery due to a scrotal hematoma that was managed conservatively. His postoperative serum creatinine fell from 1.2 to 1.0 mg/dL during his ICU stay and remained stable thereafter.

## Discussion

The VExUS scoring system was first validated in a cohort of cardiac surgical patients with an aim to detect clinically significant venous congestion [2]. Higher VExUS values were associated with an increased risk of developing AKI. The physiologic mechanism of this arises from a raised venous pressure that contributes to organ hypoperfusion by reducing perfusion pressure [5]. The VExUS system incorporates hepatic and portal vein scanning in addition to standard IVC assessment. Significant hepatic and portal vein abnormalities range from increased retrograde flow and pulsatile flow, respectively. Increased systemic congestion can lead to a worsening of these abnormalities [2,6]. A normal portal vein flow is non-pulsatile with a pulsatility index of <30%. With increasing systemic congestion, this pulsatility index can increase up to 100% leading to a visibly pulsatile portal vein. In VExUS, the scoring ranges from 0 to 3 with higher scores indicating multiple venous flow abnormalities (Table 1).

**Table 1:** VExUS grading and descriptors Grade IV IVC, >2 cm and <50% collapsibility. Hepatic vein flow abnormalities, mild: S<D wave; severe: flattened, inverted or biphasic S wave. Portal vein flow abnormalities, mild: pulsatility index 0.3-.49; severe: 0.5-1.0. Pulsatility index can be calculated as, (V_max_ - V_min_)/V_max_, where V_max _is maximum velocity and V_min_ is minimum velocity. VExUS, venous excess ultrasound score; IVC, inferior vena cava

VExUS grade	Description
1	Grade IV IVC, no hepatic vein/portal vein abnormality
2	Grade IV IVC, mild hepatic vein/portal vein abnormality
3	Grade IV IVC, severe hepatic vein/portal vein abnormality

Since its introduction, there have been multiple clinical applications of VExUS parameters. It has been used in guiding targeted fluid removal in cardiorenal syndrome, diagnosing subtypes of hyponatremia, determining the cause of AKI, predicting postoperative AKI, evaluating right ventricular dysfunction, and guiding decongestive therapy in heart failure [4,7-8]. Our case describes another potential application of VExUS where it can be used by the anesthesiologist in the perioperative period to guide therapeutic interventions in patients with advanced heart disease.

POCUS is an increasingly commonplace diagnostic modality that can provide important diagnostic information for the anesthesiologist [9,10]. The assessment of fluid status and venous congestion is usually done by assessing for IVC size and collapsibility with respiration [11]. A formal echocardiogram was not performed on this patient’s current admission as the diagnosis was already established and POCUS provided the anesthesia team with the necessary information they required for planning perioperative care.

An IVC size more than 2 cm and <50% collapsibility with no portal or hepatic flow abnormalities is classified as a grade 1 VExUS [2]. However, there are several limitations to relying solely on IVC assessment that clinicians should be aware of, which may restrict its clinical utility [3]. In our patient, an enlarged right ventricle with reduced contractility on ultrasound may have indicated the presence of pulmonary hypertension. An enlarged IVC in the presence of pulmonary hypertension may not exclude fluid responsiveness. This scenario can lead to intravascular depletion if diuretics are inadvertently used. An enlarged IVC in our patient may also reflect the chronicity of his volume overload state. Even after postoperative diuresis with a negative balance of more than 4 L, our patient still had an enlarged IVC. Hence, clinicians should be aware of these limitations to prevent relying solely on IVC for fluid assessment. The addition of portal and hepatic waveforms can provide more information to the clinician in helping to determine the intravascular state of the patient.

Portal and hepatic veins are usually interrogated using pulse wave Doppler analysis [2,4,6]. Pulse wave Doppler allows for the quantification of the flow abnormalities and in the case of the portal vein, the pulsatility index can be easily determined from the spectral waveforms. However, pulse wave Doppler may not be available in most handheld portable ultrasound devices. In severe cases of systemic congestion, as in our patient (VExUS 3), visible pulsations of the portal vein and increased retrograde hepatic vein flow can be detected on color Doppler of these vessels. On observing the color Doppler images of the portal vein in congestive states, it can appear almost arterial in nature due to its pulsatility (Video 2) whereas it is normally non-pulsatile in nature (venous flow). This can be appreciated by comparing the preoperative VExUS images of the portal vein (Video 2) to the postoperative images (Video 3). Likewise, flow abnormalities in the hepatic vein can be detected on color Doppler by the appearance of increased pulsatility and red retrograde flow as seen in Video 2. As color Doppler is available in most portable devices, this can allow the clinician to make a fairly accurate determination of a patient’s congestive state at the bedside. Solely relying on color Doppler analysis can, however, miss lower grades of dysfunction and may make it difficult to quantify congestion and assess the response to decongestive treatments.

Our patient presented with significant heart failure for an urgent procedure. His cardiovascular condition is a known risk factor for postoperative AKI and pulmonary edema [1,12,13]. Fluid shifts, pain, and the overall stress response of surgery are additional contributory factors, which can further predispose one to postoperative AKI and pulmonary edema. Excessive diuresis and hypovolemia may also be contributory factors for postoperative AKI. Despite the use of several days of diuretics, our patient was still significantly overloaded. A clinical examination or simple ultrasound assessment of his IVC may have been inadequate to fully confirm his fluid status. The addition of VExUS parameters from the ultrasound assessment of hepatic and portal veins can provide additional information that allows for a more accurate determination of the patient's congestive state.

An additional consideration of the perioperative period is the hemodynamic impact of the method of anesthesia. Excessive diuresis may have rendered him hypovolemic and unable to tolerate the vasodilation associated with the epidural anesthetic. This coupled with his essentially fixed stroke volume could have led to potentially catastrophic hypotension. With this in mind, we were hesitant to perform further diuresis before surgery. Under epidural anesthesia and with the use of the low-dose noradrenaline infusion, his hemodynamic state remained stable during the intraoperative period. Despite this stability, a discharge to the general surgical ward would have been premature. We instead opted to admit him to the ICU where we performed further diuresis guided by VExUS. This produced significant symptomatic relief, a stable postoperative creatinine, and no episodes of pulmonary edema.

Although first introduced as a prognostic tool for AKI, subsequent uses of VExUS have demonstrated its potential to be used to guide therapeutic decision-making [4,7,8]. The perioperative management of heart failure patients, as highlighted by our case is another area where VExUS can prove to be useful. A potential limitation of the use of VExUS includes its use in chronic liver disease where low hepatic pulsatility can be present even when there is significant systemic congestion [4]. Further studies in the perioperative setting, and especially in non-cardiac surgery, are necessary to further delineate its role and potential limitations.

## Conclusions

VExUS is a promising ultrasound-guided modality that can detect clinically significant organ congestion. In the perioperative setting, this can help guide decongestive therapy in heart failure patients and hence potentially reduce the risk of postoperative AKI and pulmonary edema.
